# An introduction to the Spanish version of the Mobile Toolbox

**DOI:** 10.1002/alz70857_107239

**Published:** 2025-12-24

**Authors:** Richard C. Gershon, Elizabeth M Dworak, Jiwon Kim, Miriam Novack, Sarah Pila, Y. Catherine Han

**Affiliations:** ^1^ Northwestern University Feinberg School of Medicine, Chicago, IL, USA; ^2^ Northwestern University, Chicago, IL, USA; ^3^ Northwestern University, Chicaho, IL, USA

## Abstract

**Background:**

Despite Spanish being the second most spoken language in the U.S., few cognitive assessments are available in Spanish for large‐scale studies with older adults. Culturally inclusive measures that promote early diagnosis and identify modifiable risk factors for Alzheimer's Disease and ADRD are of utmost importance. The Mobile Toolbox (MTB) provides brief, sensitive measures for assessing neurological and behavioral functions across the adult lifespan, aiding large‐scale studies on cognitive functioning and the development of ADRD. MTB integrates with the REDCap system and MyCap Mobile App, used by over 7,600 institutions in 160 countries for remote study management and delivery. The English MTB tests, released in 2024, are valid and reliable across diverse samples. In January 2025, Spanish cognitive measures were added. In this presentation, we introduce the new Spanish tests, present the Spanish Word Meaning calibration study results, and demonstrate platform usage.

**Method:**

Seven of the eight English versions of the MTB cognitive measures were deemed suitable for adaptation and developed into Spanish using a team of native Spanish speakers. When appropriate, test stimuli were updated to be more culturally sensitive. A calibration study with 1,620 Spanish‐speaking adults (Mean Age = 43.79, SD = 14.45) refined the Spanish Word Meaning item bank, crucial for developing an accurate computer adaptive test (CAT) for Spanish vocabulary.

**Result:**

The MTB library includes Spanish cognitive tests assessing language (Word Meaning), executive functioning (Arrow Matching; Shape‐Color Sorting), associative memory (Faces and Names), episodic memory (Arranging Pictures), working memory (Sequences) and processing speed (Numbers Symbol Match). The Spanish Word Meaning test used the Rasch model for consistency with its English counterpart, after removing 88 poorly fitting items. The final pool contained 515 items, with difficulty parameters ranging from ‐1.896 to 2.074.

**Conclusion:**

The MTB addresses various scientific, practical, and technical challenges in cognitive assessment by leveraging advances in technology, measurement, and cognitive research. It is suitable for a wide range of studies, including large‐scale research, clinical research, and pharmaceutical studies, particularly those interested in incorporating point‐in‐time and burst designs, as well as ecological momentary assessment (EMA). By offering tests in English and Spanish, MTB can support research with diverse populations.

